# Inherited Thrombophilia in Pediatric Venous Thromboembolic Disease: Why and Who to Test

**DOI:** 10.3389/fped.2017.00050

**Published:** 2017-03-14

**Authors:** C. Heleen van Ommen, Ulrike Nowak-Göttl

**Affiliations:** ^1^Department of Pediatric Hematology, Sophia Children’s Hospital Erasmus MC, Rotterdam, Netherlands; ^2^Thrombosis and Hemostasis Unit, Department of Clinical Chemistry, University Hospital of Kiel and Lübeck, Kiel, Germany

**Keywords:** thrombophilia, venous thromboembolism, pediatric, risk factor, counseling

## Abstract

Venous thromboembolic disease in childhood is a multifactorial disease. Risk factors include acquired clinical risk factors such as a central venous catheter and underlying disease and inherited thrombophilia. Inherited thrombophilia is defined as a genetically determined tendency to develop venous thromboembolism. In contrast to adults, acquired clinical risk factors play a larger role than inherited thrombophilia in the development of thrombotic disease in children. The contributing role of inherited thrombophilia is not clear in many pediatric thrombotic events, especially catheter-related thrombosis. Furthermore, identification of inherited thrombophilia will not often influence acute management of the thrombotic event as well as the duration of anticoagulation. In some patients, however, detection of inherited thrombophilia may lead to identification of other family members who can be counseled for their thrombotic risk. This article discusses the potential arguments for testing of inherited thrombophilia, including factor V Leiden mutation, prothrombin mutation, and deficiencies of antithrombin, protein C, or protein S and suggests some patient groups in childhood, which may be tested.

## Introduction

Venous thromboembolism (VTE) is increasingly recognized in children. In the 1990s, the annual incidence was estimated to be 0.07–0.14 per 10,000 children (1, 2). Since then, studies showed more and more children developing thromboembolic complications as a result of improved diagnosis, increased survival of children with severe underlying diseases, and increased use of invasive procedures and instruments such as central venous catheters. From 2001 to 2007, diagnosis of VTE increased from 34 to 58 cases per 10,000 hospital admissions in the United States (3). This increase was observed in all age categories. Neonates and adolescents have the greatest risk for VTE (4). It is a serious disease, which leads to mortality and morbidity. The mortality rate is about 2%. Morbidity includes lack of thrombus resolution in 50% of the patients and the development of post thrombotic syndrome in about 30% of the patients (5).

In contrast to adults, most of the venous thrombi in children are associated with clinical risk factors. In neonates, more than 90% of the thrombi are catheter related. In older children, catheters are important risk factors as well, as about 50% of VTE are associated with central venous catheters (4). In addition, other clinical risk factors contribute to the thrombotic risk, including cardiac disease, malignancy, surgery, immobility, and medications such as asparaginase and estrogen-containing contraceptives. These clinical risk factors may trigger VTE in thrombophilia carrier patients. It is still a matter of debate whether it is useful to test thrombophilia in children with a first venous thrombotic event. This article will discuss the potential arguments for testing of inherited thrombophilia, including factor V Leiden mutation (FVL), prothrombin mutation (FIIm), and deficiencies of antithrombin, protein C, or protein S and suggests some pediatric patient groups, which may be candidates for testing.

## Inherited Thrombophilia

Inherited thrombophilia is defined as an inherited coagulation disorder associated with an increased risk for thrombosis. The most frequent inherited thrombophilic defects include deficiencies of antithrombin, protein C, protein S, FVL, and FIIm.

Antithrombin deficiency was the first identified genetic risk factor for VTE. In 1965, Egeberg reported a family with increased risk for venous thrombosis due to antithrombin deficiency (6). He was the first who used the term thrombophilia. Antithrombin deficiency appears to be very scarce with a prevalence of about 0.02% in the general population (7).

Antithrombin inhibits several enzymes of the coagulation system including factor IIa, IXa, Xa, and XIIa. Type I deficiency is associated with low antithrombin antigen and activity levels. Type II deficiency is characterized by decreased functional activity. Homozygous antithrombin deficiency type I patients have not been described, assuming complete antithrombin deficiency is not compatible with life.

Protein C and protein S act together to inactivate factor Va and factor VIIIa. Protein C deficiency has been recognized since 1981 (8). Since then, more than 160 mutations have been identified. The prevalence of protein C deficiency is estimated to be about 2% (7). Two types of protein C deficiency have been described. Type 1 deficiency is associated with decreased antigenic levels as well as functional activities of protein C. In type II deficiency, the activity levels of protein C are more decreased than the antigenic levels.

Protein S deficiency has first been described in 1984 (9). The prevalence is low and varies between 0.026 and 0.13% (7). Two forms of protein S are present in the plasma: about 60% is bound to complement regulator C4b-binding protein and the remaining 40% circulates as free protein S. Only free protein S can serve as a cofactor for activated protein C. There are three types of protein S deficiency. Types I and III are quantitative deficiencies. In type I, levels of both free and total protein S are low, whereas only free protein S levels are decreased in type III deficiency. Type II deficiency is a qualitative disorder with normal antigen and decreased activity levels of protein S.

Both protein C and S deficiency can present in heterozygous, homozygous, or compound heterozygous forms, although the last two forms are extremely rare. These patients present with neonatal purpura fulminans (10, 11). In infancy, diagnosis of homozygous protein C or S deficiency can be made by measuring the functional activity of protein C or S, which will be undetectable. Diagnosis of heterozygous protein C, S, or antithrombin deficiency, however, will be challenging as all anticoagulant protein levels are physiologically lower in healthy (preterm) neonates compared to adult levels as result of developmental hemostasis (12). Protein S and antithrombin levels reach adult levels over the first 6 months of life. Protein C levels may remain low until adolescence (13).

FVL is the most common inherited thrombophilic defect although its prevalence varies widely. Prevalences of heterozygous FVL range from 1 to 9% in European countries, whereas it is rarely found in African and Asian individuals (14). FVL is characterized by a substitution of glutamine by arginine on position 506 of the factor V protein at the activated protein C cleavage site. It causes resistance of factor Va to cleavage by activated protein C leading to an excess of factor Va and, consequently, a hypercoagulable state (15, 16).

FIIm is the second most common inherited thrombophilic defect. In Caucasians, the prevalence of this mutation is about 2% (17). It is characterized by a point mutation (nucleotide 20210 G to A) in the prothrombin gene, which is associated with increased levels of prothrombin, the precursor of thrombin (18). Increased levels of prothrombin increase the half-life of factor Va. As factor Va, bound in the prothrombin-factor Va complex, is resistant to activated protein C cleavage, increased levels of prothrombin increase the half-life of factor Va, leading to a hypercoagulable state.

## Why Should We Test for Inherited Thrombophilia in Children with a First Venous Thrombotic Event?

In general, there are three possible arguments to test for thrombophilia in children with a first venous thrombotic event. First, if there is an association between inherited thrombophilia and the development of pediatric thrombosis, identification of a thrombophilic defect may help to learn why a young patient developed thrombosis, especially if the thrombotic event was unprovoked. Second, testing should be performed if a positive test result will change the patient’s management, such as prolongation of anticoagulant prophylaxis of recurrent thrombotic events. Finally, testing pediatric patient with VTE may help to identify asymptomatic relatives who may avoid thrombotic risk factors and benefit from thromboprophylaxis in high-risk situations.

### Association between Inherited Thrombophilia and Venous Thrombosis

Patients, parents, and their doctors would often like to have an explanation for the VTE event. Several case series, case-control studies, registries, and cohort studies have been published, which studied the impact of inherited thrombophilia on VTE in children. In 2008, Young et al included these studies in a meta-analysis, which showed that children with first-onset VTE were more likely to have inherited thrombophilia than controls (19). The odds ratios varied from 2.63 [95% confidence interval (CI), 1.61–4.29] for FIIm to 8.73 (95% CI, 3.12–24.42) for antithrombin deficiency. These ORs resembled the relative risks found in adults with VTE (20). So, in general inherited thrombophilia contributes to the development of VTE in children. Testing for thrombophilia might reveal one of the causes of the thrombotic event in a pediatric patient. The identification of a thrombophilic defect, though, does not exclude other risk factors as shown in the meta-analysis. More than 70% of the patients had at least one clinical risk factor, illustrating that a thrombophilic defect alone is usually not enough to develop pediatric thrombosis.

The association between inherited thrombophilia and certain patient subgroups is less clear. An important limitation of the above-mentioned meta-analysis was that patient subgroups like provoked or unprovoked, neonatal VTE, and catheter-related VTE could not be analyzed separately due to small groups, and unclear definitions in the original studies. Nevertheless, other studies showed that the prevalence of inherited thrombophilia seems to be higher in adolescents with unprovoked thrombosis and in children with a positive family history for VTE and lower in children with cardiac disease or malignancy with catheter-related thrombosis (21–24). In neonates with catheter-related thrombosis, only a few small studies investigated the prevalence of thrombophilia defects, which were rarely found (25–27).

### Thrombophilia and the Management of Thrombosis

A more important reason in favor of thrombophilia testing would be the need to adjust the management of thrombosis in case of a positive result. At the moment, the identification of an inherited thrombophilic defect does not alter the acute antithrombotic management in children (28). Whether the duration of anticoagulation will change after discovery of a thrombophilic defect is dependent on the risk of side effects of anticoagulation, such as major bleeding, the risk of recurrent VTE and the preference of the patient. In adults, the annual incidence of major bleeding from long-term anticoagulation is 1.5–2% (29, 30).

Generally, the cumulative recurrence-free survival in children is reported to be 92% after 1 year and 82% after 7 years of follow-up (24). Young et al. studied the association between inherited thrombophilia and recurrent VTE in children (19). Their meta-analysis showed a significant but mild association for all thrombophilic defects, except FVL. The ORs varied from 2.15 (95% CI, 1.12–4.10) for FIIm to 3.76 (95% CI, 1.76–8.04) for protein S deficiency. Children with two or more thrombophilic defects had the highest risk for recurrent VTE (OR 4.91; 95% CI, 3.12–7.74). The thrombotic recurrence risk in children with thrombophilia was slightly higher than that in adults (Table 1). This might be caused by lack of prophylactic anticoagulation in high-risk situations after the first thrombotic event. In children, it was not common practice to administer thromboprophylaxis in high-risk situations such as immobility, surgery, or trauma. In adults as well as in children, the mild increased recurrence risk in patients with inherited thrombophilia has not lead to adjustment of the duration of anticoagulant therapy.

**Table 1:** **Association-inherited thrombophilia with risk of VTE in children**.

	Prevalence (%) (population)	Summary OR (95% CI) first VTE (19)	Summary OR (95% CI) recurrent VTE (19)	Annual risk (%, 95% CI) for recurrence after non-CVC-related VTE (31)	Annual risk (%, 95% CI) for VTE in asymptomatic carriers^a^ (32)	
Antithrombin deficiency	0.02	8.73 (3.12–24.42)	3.37 (1.57–7.20)	5.4 (2.6–10)	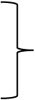	2.82 (1.63–4.80)
Protein C deficiency	0.2	7.75 (4.48–13.38)	2.53 (1.30–4.92)	1.3 (0.3–3.8)		
Protein S deficiency	0.03–0.13	5.77 (3.07–10.85)	3.76 (1.57–8.04)	0.7 (0.08–2.4)		
Factor V Leiden	3–7	3.56 (2.57–4.93)	0.77 (0.40–1.45)		0.25 (0.12–0.53)	
Prothrombin mutation	1–2	2.63 (1.61–4.29)	2.15 (1.12–4.10)		0.42 (0.17–1.01)	

*^a^First- or second-degree relative of pediatric patient with venous thromboembolism (VTE) and thrombophilia*.

*OR, odds ratio; CI, confidence interval; CVC, central venous catheter*.

Very recently, Limperger et al. studied the annual recurrence rate of pediatric patients after a first non-catheter-related VTE (31). In general, the estimated risk of VTE recurrence was 1.5% per year. In children with high-risk thrombophilia, the annual recurrence rates were 5.4% (95% CI, 2.6–10%) in children with antithrombin deficiency, but only 1.3% (95% CI, 0.3–3.8%) and 0.7% (95% CI, 0.08–2.4%) in protein C and protein S deficient patients, respectively. In patients with no thrombophilia, the annual recurrence rate was 0.9% (95% CI, 0.4–1.8%). Thus, based on these results, particularly antithrombin-deficient patients have an increased recurrence risk. These patients might be identified by testing and benefit from preventive strategies.

These preventive strategies may include indefinite anticoagulation with vitamin K antagonists or intermittent anticoagulation in high-risk situations. Both strategies have not been studied in children yet. As in the study of Limperger et al., 9 out of the 10 children with antithrombin deficiency had provoked recurrent VTE, the latter strategy with intermittent anticoagulation might be sufficient enough to prevent recurrent VTE with less risk of bleeding. One might argue, however, that every young patient should get intermittent prophylactic anticoagulation in high-risk situations after a first VTE, independent from the presence of inherited thrombophilia. Due to decreased bleeding risk, the new direct oral anticoagulants might appear to have a favorable benefit-to-risk ratio for prolonged anticoagulation in children with antithrombin deficiency in future.

### Identification of Asymptomatic Relatives

It is suggested that inherited thrombophilia testing in pediatric patients with VTE allows the identification of asymptomatic family members with thrombophilia. The affected relatives will have the opportunity to avoid risk factors such as smoking and obesity to get informed about thrombotic risks of contraception and pregnancy and to use thromboprophylaxis in high-risk situations.

Holzhauer et al. investigated the general, annual incidence of VTE in first- and second-degree relatives of pediatric patients with VTE and inherited thrombophilia (32). The absolute risk of a first thrombotic event per year in asymptomatic relatives was not very high. It was higher in carriers of antithrombin, protein C or S deficiency (2.82%; 95% CI, 1.63–4.80%) than in carriers of FIIm or FVL (0.42%; 95% CI, 0.17–1.01% and 0.25%; 95% CI, 0.12–0.53%), respectively. In relatives without inherited thrombophilia, the absolute VTE risk per year was 0.10% (95% CI, 0.06–0.17%). As in adults, the inherited thrombophilic defects can, therefore, be divided in low-risk thrombophilia, including FVL and FIIm and high-risk thrombophilia, including deficiencies of antithrombin, protein C or S (32, 33). Remarkably, almost 65% of VTE in the first- and second-degree relatives of pediatric VTE patients with thrombophilia in the study of Holzhauer et al. was associated with clinical risk factors. Thus, discussion with asymptomatic relatives about avoidance of lifestyle risk factors such as obesity and smoking and eventually thromboprophylaxis in high-risk situations might be enough to prevent most VTE, regardless of inherited thrombophilia status.

Screening has been recommended for adolescents at fertile age with a family history of thrombosis and/or thrombophilia before start of oral contraceptives (34). Combined oral contraceptives are an important risk factor for VTE. The thrombotic risk is much higher in women with high-risk than with low-risk thrombophilia. The annual thrombotic risk on combined oral contraceptives is 4.3% for asymptomatic carriers with a positive first-degree relative with VTE and high-risk thrombophilia. The risk is only 0.2–0.5 per year of use for asymptomatic carriers with a positive first-degree relative with VTE and low-risk thrombophilia (35). As a consequence, it may be worthwhile to test adolescents from families with high-risk thrombophilia to avoid use of combined contraceptives. However, in case of strong family history for VTE, combined contraceptives should be avoided in asymptomatic carriers of low thrombophilia, as well. When counseling these patients, it is important to realize that in families with a family history for VTE and/or thrombophilia, the thrombotic risk is also increased in unaffected relatives, due to yet unknown genetic variables and/or clinical risk factors (35). Consequently, negative thrombophilia testing may cause false reassurance. Therefore, some guidelines advise to consider an alternative contraceptive in women with a first-degree relative with VTE, independent of testing (36).

## Disadvantages of Testing

One of the disadvantages of testing children with VTE could be the psychological stress of knowing to be a carrier of an inherited thrombophilic defect. No studies are available investigating the psychological impact of thrombophilia testing on children. In adults, thrombophilia testing does not seem to trigger psychological stress or worry (37, 38). Another disadvantage may be the potential problems with health insurance or life insurance in future. One of the patients in the study of Bank et al. had been discriminated by an insurance company because of FVL. The other 16 patients had not informed the insurance companies (39). Finally, thrombophilia testing is expensive. Many tests are ordered inappropriately (40, 41). Proper thrombophilia testing will reduce costs.

## Who Should We Test for Inherited Thrombophilia in Children with a First Thrombotic Event?

As mentioned before, the chance of finding inherited thrombophilia varies between pediatric patient groups with VTE. Inherited thrombophilia seems to be present in adolescents with unprovoked thrombosis and children with a positive family history. In the study of Revel-Vilk et al., thrombophilia was detected in only 13% of all 171 pediatric patients with VTE, but in 60% of the adolescents with unprovoked thrombosis (22). Ruud et al. showed in a cross-sectional study that a positive family history for VTE increased the relative risk of a child having inherited thrombophilia to 2.35 (95% CI, 1.1–5.2) (42). In addition, in a prospective cohort study of 100 neonates and children with VTE, positive family history appeared to be the only predictor for presence of inherited thrombophilia (OR 14.9; 95% CI, 1.9–113) (24). In neonates and children with catheter-related thrombosis, thrombophilia was found less frequently. For example, Salonvaara et al. described 10 neonates with symptomatic catheter-related thrombosis. Only 1 of the 10 patients had FVL (26). Albisetti et al. studied the prevalence of thrombophilia in cancer patients with and without catheter-related thrombosis. Thrombophilia was found in 4% of cancer patients with VTE and in 12% of patients without VTE (21). Finally, Thom et al. could not find an association between catheter-related VTE and thrombophilia in cardiac pediatric patients (23).

So, thrombophilia testing seems to be advisable in adolescents with unprovoked VTE and in children with a positive family history for VTE and less useful in neonates and children after a first episode of catheter-related VTE.

## Summary

The presence of inherited thrombophilic defects in neonates and children with a first event of VTE does not influence primary antithrombotic management and while it may be one of the causes of the thrombotic event, it infrequently alters long-term management. However, the presence of antithrombin deficiency with its high risk of recurrent thrombosis may warrant adjustment of long-term management. In these children, intermittent anticoagulation in high-risk situations may aid to prevent recurrent thrombotic episodes. Furthermore, testing might be helpful to identify asymptomatic female relatives with thrombophilia who may get better informed about a healthy lifestyle and the risks of combined oral contraceptives at fertile age.

On the other hand, one might argue that all children with first VTE are candidates for prophylactic anticoagulation in high-risk situations, not only antithrombin deficient children. And a positive family history for VTE might be enough to choose less thrombotic contraceptive methods, such as progesterone-only preparations (Table 2).

**Table 2:** **Arguments pro and contra thrombophilia testing in children with venous thromboembolism (VTE)**.

Arguments pro thrombophilia testing	Arguments contra thrombophilia testing
**Association between inherited thrombophilia and VTE in children**	
Explanation of pathophysiology, especially if VTE was unprovoked	Most pediatric patients have at least one clinical risk factor, illustrating that a thrombophilic defect alone is not enough to develop VTE

	It is unclear whether the association is valid for all patients groups, for example, children with catheter-related thrombosis

**Management of VTE in children**	
Prediction of recurrence risk and opportunity for prophylactic anticoagulation in high-risk situations, especially in patients with antithrombin deficiency	All children should get prophylactic anticoagulation in high-risk situations after first VTE

	Efficacy of prophylactic strategies have not been studied in children

**Identification of asymptomatic relatives with inherited thrombophilia**	
Instruction about signs and symptoms of VTE to accelerate diagnosis and avoidance of thrombotic risk factors, such as obesity and smoking	Testing is not necessary to instruct patient and family members about signs and symptoms of VTE and to avoid thrombotic risk factors

Opportunity for prophylactic anticoagulation in high-risk situations, especially in patients with high-risk thrombophilia	As most VTE in affected family members are provoked, thromboprophylaxis in high-risk situations might be enough to prevent VTE

Counseling about combined oral contraceptives in asymptomatic female carriers with thrombophilia	False reassurance if thrombophilia testing is negative

	Consider alternative contraceptive in all women with first-degree relative with VTE, without testing

	In general: psychological distress of knowing to be a carrier and difficulties to obtain health or life insurances

Consequently, testing for inherited thrombophilia in children with a first episode of VTE should not be performed on a routine basis. In neonates and children with catheter-related thrombosis, inherited thrombophilia seems to contribute less to the thrombotic event than in adolescents with unprovoked thrombosis and children with a positive family history for VTE. Therefore, especially these latter groups seem candidates for testing.

In these children, the decision of testing should be made on an individual base after proper counseling before and after testing by an experienced physician, discussing the disadvantages and benefits of testing, the preference of the patient, and the consequences of the test results after testing.

## Author Contributions

CO and UN-G were both involved in drafting the concept of the manuscript. CO wrote the manuscript, UN-G read, edited, and approved final manuscript.

## Conflict of Interest Statement

The authors declare that the research was conducted in the absence of any commercial or financial relationships that could be construed as a potential conflict of interest.

## Abbreviations

VTE, venous thromboembolism; FVL, factor V Leiden mutation; FIIm, prothrombin mutation; CI, confidence interval.
